# Human IgG antibody responses to severe acute respiratory syndrome coronavirus 2 viral antigens receptor-binding domain, spike, and nucleocapsid, in vaccinated adults from Merida, Mexico

**DOI:** 10.3389/fmed.2022.916241

**Published:** 2022-07-22

**Authors:** Henry Puerta-Guardo, Manuel Parra-Cardeña, Fernando Peña-Miranda, Felipe Flores-Quintal, Pilar Granja-Pérez, Salha Villanueva-Jorge, Refugio González-Losa, Laura Conde-Ferraez, Jesus Gómez-Carballo, Gonzalo Vazquez-Prokopec, James T. Earnest, Pablo Manrique-Saide, Guadalupe Ayora-Talavera

**Affiliations:** ^1^Centro de Investigaciones Regionales Dr. Hideyo Noguchi, Universidad Autónoma de Yucatán, Mérida, Mexico; ^2^Unidad Colaborativa para Bioensayos Entomológicos, Campus de Ciencias Biológicas y Agropecuarias, Universidad Autónoma de Yucatán, Mérida, Mexico; ^3^Laboratorio Estatal de Salud Pública, Servicios de Salud de Yucatán, Mérida, Mexico; ^4^Department of Environmental Sciences, Emory University, Atlanta, GA, United States

**Keywords:** SARS-CoV-2 antigens, vaccinees, IgG response, Pfizer, CanSino

## Abstract

Several vaccines against severe acute respiratory syndrome coronavirus 2 (SARS-CoV-2) have been approved for controlling the coronavirus disease 2019 (COVID-19) pandemic worldwide. Antibody response is essential to understand the immune response to different viral targets after vaccination with different vaccine platforms. Thus, the main aim of this study was to describe how vaccination with two distinct SARS-CoV-2 vaccine preparations elicit IgG antibody specific responses against two antigenically relevant SARS-CoV-2 viral proteins: the receptor-binding domain (RBD) and the full-length spike (S). To do so, SARS-CoV-2 protein specific in-house enzyme-linked immunosorbent assays (ELISAs) were standardized and tested against serum samples collected from 89 adults, recipients of either a single-dose of the Spike-encoding mRNA-based Pfizer/BioNTech (Pf-BNT) (70%, 62/89) or the Spike-encoding-Adenovirus-5-based CanSino Biologics Inc. (CSBIO) (30%, 27/89) in Merida, Mexico. Overall, we identified an IgG seroconversion rate of 88% (68/78) in all vaccinees after more than 25 days post-vaccination (dpv). Anti-RBD IgG-specific responses ranged from 90% (46/51) in the Pf-BNT vaccine at 25 dpv to 74% (20/27) in the CSBIO vaccine at 42 dpv. Compared to the S, the RBD IgG reactivity was significantly higher in both Pf-BNT (*p* < 0.004) and CSBIO (*p* < 0.003) vaccinees. Interestingly, in more than 50% of vaccine recipients, with no history of COVID-19 infection, antibodies against the nucleocapsid (N) protein were detected. Thus, participants were grouped either as naïve or pre-exposed vaccinees. Seroconversion rates after 25 and more dpv varies between 100% in Pf-BNT (22/22) and 75% (9/12) in CSBIO pre-exposed vaccinees, and 89% (26/29) and 73% (11/15) in Pf-BNT and CSBIO naïve vaccine recipients, respectively. In summary, observed seroconversion rates varied depending on the type of vaccine, previous infection with SARS-CoV-2, and the target viral antigen. Our results indicate that both vaccine preparations can induce detectable levels of IgG against the RBD or Spike in both naïve and SARS-CoV-2 pre-exposed vaccinees. Our study provides valuable and novel information about the serodiagnosis and the antibody response to vaccines in Mexico.

## Introduction

The coronavirus disease 2019, better known as COVID-19, is an ongoing pandemic caused by severe acute respiratory syndrome coronavirus 2 (SARS-CoV-2) (1). The first known COVID-19 case was identified in December 2019 in Wuhan, China (2, 3). Since then, SARS-CoV-2 has rapidly spread around the world causing over 340 million cases and claimed over 5 million lives worldwide (1). The COVID-19 pandemic has posed an extraordinary threat to the global public health, and the global economy (4, 5).

Severe acute respiratory syndrome coronavirus 2 is an enveloped, single-positive-strand RNA virus belonging to the β-coronavirus genus (6, 7). The coronavirus genome encodes 4 major structural proteins: spike (S), envelope (E), membrane (M), and nucleocapsid (N), and approximately 16 non-structural proteins (nsp1–16), and 5–8 accessory proteins (2, 7, 8). During infection with SARS-CoV-2, the structural proteins, S and N, constitute the main targets to generate antibodies that neutralize viral particles and prevent infection of host cells (8–13). These antibody responses have shown different times for seroconversion against distinct viral antigens depending on the severity of the disease (14–16). The S and N proteins have shown to be highly immunogenic, being the S the main target for neutralizing antibodies (6, 8, 9, 17). While protective antibodies can potentially bind a large portion to the S protein, for SARS-CoV-2 the receptor-binding domain (RBD) is especially important as it interacts with the host cell receptor, the angiotensin converting enzyme 2 (ACE2) resulting in virus entry and infection. Thus, the RBD within the S represents a critical target when looking at humoral immune responses (18–20). Importantly, neutralizing antibodies against the RBD have been widely studied and shown to be effective in SARS-CoV-2 protection *in vitro* and *in vivo* (9, 20, 21). Most of the antibodies targeting other structural proteins such as N do not have neutralizing activity against SARS-CoV-2 infection; however, they have been reported to be highly useful for diagnosis and epidemiology purposes (22–24).

Several vaccine candidates, mainly directed against the S protein of SARS-CoV-2, have been approved by the World Health Organization (WHO) Strategic Advisory Group of Experts on Immunization (SAGE) for emergency use (25). Since December 2020, 10 of these WHO-approved vaccines have been deployed and administrated in more than 64% (>77 million people) of the population of Mexico. These include one protein subunit-based vaccine (CIGB-66) by Center for Genetic Engineering and Biotechnology (CIGB); two mRNA-based vaccines by Moderna (mRNA-1273) and Pfizer/BioNTech (Pf-BNT) (BNT162b2, aka Comirnaty); four non-replicating viral vector by CanSino (Ad5-nCoV, aka Convidencia), Gamaleya (Gam-COVID-Vac, Sputnik V), Johnson & Johnson (Janssen, Ad26.COV2.S), and Oxford/AstraZeneca (Vaxzevria, ChAdOx1 nCoV-19 or AZD1222); and three virus inactivated-based vaccines by Bharat Biotech (Covaxin), Sinopharm-Beijing (Covilo, BBIBP-CorV), and Sinovac (CoronaVac) (26, 27).

The COVID-19 disease may course from asymptomatic to symptomatic mild and sometimes life-threatening complications such as the acute respiratory distress syndrome (ARDS) (3, 4). Several studies have estimated a wide range of asymptomatic infections between 4 and 80% (28–33). As SARS-CoV-2 continues spreading globally with new viral variants emerging, and with many patients without any symptoms that can still transmit the virus, understanding the dynamics of the immune responses after natural infection or vaccination against SARS-CoV-2 becomes a critical need for public health systems worldwide. Most COVID-19 serological assays identify serum antibodies focused on two viral structural proteins, the S and N proteins (10, 12, 14, 15, 21, 23, 34, 35). However, the time it takes to develop detectable antibodies against these proteins has been shown to vary based on disease severity after natural infection (14–16). Antibodies against the S protein of SARS-CoV-2 are not normally detected at early days of infection (from day 0 to day 3), and peaks at day 25. On the other hand, seroconversion against the N protein seems to happen faster as early as 3 days after illness onset, being a good marker of a more recent infection (12, 35–39). Despite this, few studies have addressed the dynamics of anti-SARS-CoV-2 IgG responses following vaccination in Mexico (40).

Here, we measure the IgG-specific responses in vaccinated individuals against three main SARS-CoV-2 viral targets, using standardized in-house indirect enzyme-linked immunosorbent assays (ELISAs). We report seroconversion and variable IgG reactivity against the three viral targets, RBD, S, and N after vaccination with two distinct SARS-CoV-2 vaccines, an mRNA-based vaccine Pf-BNT and CSBIO.

## Materials and methods

### Ethics statement and study approval

This study was approved by the Ethics Committee Board of the Research Center “Dr. Hideyo Noguchi” of the Autonomous University of Yucatan (CIR-UADY) (Protocol number: Record CEI-11-2020) and the Ethics Commission of the State Laboratories of the Public Health Services of Yucatan. All participants provided verbal understanding and completed a written informed consent.

#### Study participants and collection of human samples

Between April and June 2021, a total of 89 adult volunteers were enrolled into a prospective observational study led by the Virology Laboratory at CIR-UADY. Participants were employees of UADY and SSY and had been previously vaccinated through the National Immunization Program against COVID, with a single dose of the mRNA-based vaccine Pf-BNT (*n* = 62) or the CSBIO (*n* = 27).

A total of 140 serum samples (5 mL of whole-blood) were collected by venipuncture using golden-cap tubes (SST™ 13 mm × 100 mm, BD Vacutainer) and sterile-non-pyrogenic needles (21G, Greiner bio-one). In 113 participants, serum samples were collected from the Pf-BNT group after 5 (*n* = 62) and 25 (*n* = 51) days post-vaccination (dpv). Although no basal serum samples were collected before the first dose of the Pf-BNT vaccine, a sample at 5 dpv was taken as the earliest post-vaccination time point. Also, by the time a second sample was collected at 25 dpv, participants had not received their second dose of the Pf-BNT vaccine, as it was administrated by the government after more than 40 days from the first dose.

The remaining 27 serum samples belonged to the CSBIO vaccinees. These samples were collected from vaccinees that voluntarily attended to the Virology Laboratory at CIR-UADY to be tested for anti-SARS-CoV-2 IgG antibodies at variable dpv ranging from 30 to 57 days. All serum samples were processed under biosafety level A2, and heat inactivated at 56°C for 1 h prior to short-term storage at 4°C or long-term storage at −80°C following standard protocols for sampling and handling human blood samples established at the Virology Laboratory CIR-UADY. Basic data (age, sex, and date of vaccination) were collected from each participant. All participants were residents of the city of Merida, Yucatan, Mexico.

#### Severe acute respiratory syndrome coronavirus 2 protein antigens

Protein expression and purification was performed following standard protocols as previously described by Stadlbauer et al. (21) and Byrum et al. (41) with some modifications. The viral proteins RBD, S, and N are based on the genomic sequence of the Wuhan-Hu-1 isolate (2). Plasmids pCAGGS encoding SARS-CoV-2 Spike (with a C-terminal hexa-histidine tag), and the RBD genes (with a C-terminal hexa-histidine tag) were obtained from a donation of Dr. Florian Krammer (Department of Microbiology, Icahn School of Medicine at Mount Sinai, NY, United States) (21). Plasmid pET-28 vector (41) encoding SARS-CoV-2 N gene was donated by Dr. Eva Harris and Dr. Scott Biering (Division of Infectious Diseases and Vaccinology University of California, Berkeley, CA, United States).

All SARS-CoV-2 protein-encoding plasmids were initially amplified by transforming chemically competent *Escherichia coli* DH5α cells using approximately 100 ng of purified plasmid (QIAGEN Plasmid Midi Kit, United States) grown in LB medium supplemented with Ampicillin (1 μg/mL). PCR positive colonies were grown for mass production, and plasmids were recovered and purified following manufacturer’s protocols for standard DNA plasmid purification (Zippy™ Plasmid Midiprep Kit, ZYMO Research).

#### Cell cultures, reagents, antibodies, and references sera

Expi293F™ cells were maintained following the manufacturer’s instructions under standard culture conditions of 8% CO_2_ and 37°C on an orbital oscillation platform (100–120 rpm) using Expi293 Expression Medium (Gibco #A1435102). A mouse anti-6X His-tag^®^ monoclonal antibody ([HIS.H8], Abcam 18184) and Peroxidase Affinity Pure Goat Anti-Mouse IgG (H + L) (Jackson Immuno Research # 115-035-003) was used for ELISA confirmation of the three recombinant proteins and western blot analyses. Anti-Human IgG (Fc specific)–peroxidase antibody produced in goat (Sigma, #A0170) was used for detection of SARS-CoV-2 IgG-specific antibodies present in human sera. Bovine serum albumin was used to block ELISA plates (Sigma, A9647-100G). Opti-MEM^®^ (1×) reduced serum medium for transfection experiments. Non-fat Omniblock skimmed milk (#AB101009, Americanbio) was used for blocking ELISA and western blot. Microtiter plates (Immunolon 4 HBX, Ultra-high binding polystyrene microtiter plates) for ELISA. A reference negative control serum (Accurun^®^ 810 Multi-Marker, 2017-11-11, Lot: 10087801, Seracare, United States) was used as a negative control.

#### Recombinant production of antigens

Purified plasmids were used to transfect high density (4–5 × 10^6^ viable cells/mL) cultures of suspension-adapted human embryonic kidney (HEK) cells (Expi293F™ cells, Thermo Scientific Inc., kindly donated by Dr. Jesus Hernandez at the Immunology Laboratory of the Research Center for Food and Development, Mexico) using the ExpiFectamine™ 293 Transfection Kit (Gibco, A14524) and the Expi293 Expression Medium supplemented with GlutaMAX™ following the manufacturer’s instructions. Cell-free supernatants containing soluble SARS-CoV-2 proteins were harvested and concentrated using Amicon^®^ Ultra-15 centrifugal filters with a 100 kDa cut-off for the full-length S [∼190 kDa molecular weight (mw)] and the N (∼114 mw), or the 10 kDa cut-off filters for the RBD (∼30 kDa mw) and purified following standard protocols for His-tagged protein purification using Ni-NTA agarose (QIAGEN) packed on polypropylene columns (QIAGEN), and imidazole (Sigma) for washing and elution buffers (21). Eluted proteins were buffer exchanged into sterile PBS using either Amicon Filters with 10 kDa mw cut-off for the RBD or 100 kDa for S or N and quantified using a standard Biuret Protein Assay with BSA as standard protein, then stored at −80°C until further use.

#### Severe acute respiratory syndrome coronavirus 2 IgG indirect enzyme-linked immunosorbent assay

**FIGURE 1:**
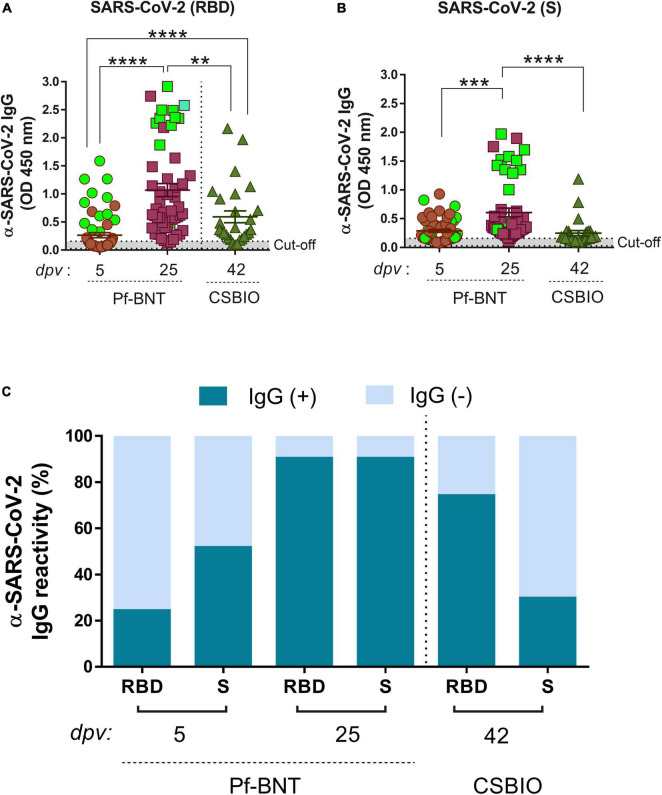
IgG-specific responses to the RBD, S, and N proteins of SARS-CoV-2 in two groups of vaccinees. IgG levels expressed as OD values against RBD **(A)**, and S **(B)**, proteins determined from positive and negative serum samples [here expressed as IgG reactivity % (*y*-axis)] based on previously established cut-off values >0.20 (horizontal dotted line and *gray zone*) **(C)**. Serum samples for Pf-BNT and CSBIO vaccinees were collected after 5 (*n* = 62) and 25 (*n* = 51), and 43 (*n* = 27) dpv, respectively [vaccine values separated by vertical dotted line (*x*-axis)]. Mann–Whitney test: ****p < 0.0001; **p < 0.001; *p < 0.05. n.s., non-significant differences. **(A,B)** Each group of data in the scatter plots represent absorbance values (OD) obtained from individual serum sample per vaccine group including the mean ± SEM (standard error) of individual groups. Participants with record of COVID-19 (PCR-positive test) before vaccination are highlighted in light green. **(C)** Stacked bars represent the percentage of serum samples per vaccine group showing IgG positive (+, *green*) or negative (–, *light blue*) OD values against individual SARS-CoV-2 viral targets. dpv, days post-vaccination.

Three ELISAs were standardized for detection and titration of human IgG antibodies to the RBD, the S, and the N proteins. Indirect ELISA protocols were adapted from Stadlbauer et al. (21), which used a final concentration of 2 μg/mL to sensitize the ELISA plates. Here, following this standard protocol, we initially optimized a final concentration of 1 μg/mL [*R*^2^ = RBD (0.9727); S (0.9573), N (0.9775)] of each antigen to coat the ELISA plates (Figure 1A). Each R square denotes the linearity of different absorbance values obtained from each ELISA curve. Later, based on the differential mw of these three SARS-CoV-2 proteins, RBD (∼25–27 kDa), N (∼45 kDa), and S (∼180 kDa), we adjusted all SARS-CoV-2 proteins concentrations into a similar molar concentration of 37 nM, finally used to sensitize each ELISA as follows: RBD (1 μg/mL), N (1.6 μg/mL), and S (6 μg/mL).

Baseline absorbance for all three viral antigens was derived from different antigen concentrations (range 10–0.78 μg/mL) in a repeatability test (*n* = 6) with the reference negative serum (dilution 1:100) and a set of 88 human sera (dilution 1:100) collected before the pandemic in 2016 (Supplementary Figure 1B). A total of 546 ELISA wells were processed against the targets, RBD, S, and N, using the reference negative serum and the 2016-sera, finding an average OD value of 0.092 (Min: 0.054; Max: 0.141). No statistical differences were identified between OD values obtained after testing these negative sera against the RBD (*n* = 182 wells), S (*n* = 182 wells), or the N (*n* = 182 wells) either among all groups (one-way ANOVA, Alpha; 0.05; CV = 0.172, 95% CI) or within individual groups (Student *t* distribution *p*-value = 0.1047) (Supplementary Figures 1B,C). Regarding the pool positive sera, variable OD values were obtained when testing for the RBD (avg. 2.25; Min: 1.77; Max: 3.20; *n* = 48), S (0.58; Min: 0.39; Max: 0.93; *n* = 104), and the N (0.41; Min: 0.21; Max: 0.77; *n* = 88), but not significant (Supplementary Figure 1D). Based on these set of data, we established an absorbance OD threshold (cut-off) value >0.20 (avg. OD = 0.092 + 3xSD) (Supplementary Figures 1B–D).

Briefly, 96-well ELISA plates (Immunolon^®^ 4 HBX, Ultra-high binding polystyrene microtiter plates) were coated overnight at 4°C with 50 μL/well of individual SARS-CoV-2 antigens at approximately 37 nM (final concentration) in sterile Phosphate Buffered Saline (PBS 1×, pH 7.4, Gibco). The next day, plates were washed in PBS and blocked for 1 h at 37°C using a 100 μL of blocking buffer (BB) containing 2% BSA/0.05% Tween-20 in PBS. Then, 50 μL of twofold serially diluted human serum (1:100 starting dilution) in diluent buffer (1% non-fat dry milk in PBS-T 0.05%), was added to each well and incubated at room temperature (RT) for 30 min in gentle oscillation. Plates were washed five times using PBS-Tween 20 (PBS-T 0.1%). The IgG titer was determined using 50 μL per well of a secondary mouse anti-human IgG monoclonal antibody HRP labeled, diluted 1:10,000 in PBS-T. Enzymatic reaction was detected with HRP-substrate (TMB, Sigma). Color development was stopped by adding 50 μL of 1N hydrochloric acid (HCL) and absorbance values were recorded at 450 nm using a microplate reader (Victort X3, 2030 multilabel reader, PerkinElmer). Pooled anti-SARS-CoV-2 positive human sera (*n* = 5) determined by the commercial ELISA kit (EDI™ Novel Coronavirus COVID-19 IgG ELISA kit, Epitope Diagnosis, OD ≥ 2.0) (11), were used to prepare a standard serum for each antigen. This positive control pool was used at 1:100 dilution throughout the study.

To discriminate amongst positive and negative results, a cut-off value was estimated using known independent negative sera (Accurun^®^ 810 Multi-Marker), along with the pool positive human sera (see above). To set up the cut-off value we used the formula (42): $C\,u\,t-o\,f\,f\,v\,a\,l\,u\,e=a.\bar{x}+f.S\,D$, where ***$\hat{x}$*** is the mean and SD the standard deviation of independent negative control readings, and ***a*** and ***f*** two multipliers. Based on this formula, we arbitrarily set an *a* = 1 with *f* = 3 (i.e., cut-off = mean +3 times the SD). The magnitude of the IgG response against SARS-CoV-2 antigens from vaccinees was defined by determining the area under the curve (AUC) for all dose-response curves, considering that all peaks above the base (cut-off) line when OD ≥ 0.20. Additional analyses were performed to identify the endpoint dilution titer, defined as the concentration required for three times the background signal of the negative sera, and the relative binding of IgG, expressed as the reciprocal of the endpoint serum dilution that results in 50% of IgG binding to the target protein measured at the high dilution tested for all samples.

### Data collection and statistical analysis

All statistical analyses and graphs were performed and generated using GraphPad Prism 6 software (GraphPad Prism 6.07). Overall, Student’s *t*-test, one-way ANOVA and unpaired non-parametric tests were used to evaluate differences between two or more groups and individual groups, respectively. Statistically significant differences among means were considered as *p*-values < 0.05. For dose-response curves, a one-way ANOVA with multiple comparisons was used to determine significance between different serum dilutions. EC_50_ values were calculated from Log_10_ normalized data followed by non-linear regression fit analyses with sigmoidal dose response (variable slope) equation of Prism 6. The mean EC_50_ and Hill slope values of the curves with 95% confidence intervals (95% Cis) were determined. Similarly, the AUC was calculated using Prism. Endpoints were compared within group but between days or doses by Wilcoxon signed rank test (Wilcoxon) for repeated measurements without normal distribution. Paired analyses of EC_50_ values were performed by *t*-test (Mann–Whitney test) with significant differences of *p* < 0.05. One sample *t*-test analysis was performed to found significant differences within each study group of data (significant, alpha = 0.05). Linear regression analysis for dose-response curves was also performed.

## Results

Study participants were 63.3 % female (59/89) and 33.7% male (30/89), either vaccinated with a single dose of the Pf-BNT vaccine (69.7%, 62/89) or the CSBIO vaccine (30.3%, 27/89). The age of participants varied between 20 and 63 (avg. 42) and 23 and 59 (avg. 43) for Pf-BNT or CSBIO vaccinees, respectively. Of those who received the Pf-BNT vaccine, 25.8% (16/62) reported mild (non-severe) symptoms of COVID-19 with a confirmatory PCR positive test before vaccination. The remaining vaccine recipients did not report any history of symptomatic infection before vaccine administration. From the total of 140 serum samples, 80.7% belonged to volunteers receiving the Pf-BNT vaccine and the remaining 19.3% received the CSBIO (Table 1).

**TABLE 1:** Demographics features of enrolled participants vaccinated against COVID-19 including type of vaccine, days post-vaccination, sex, age, and previous reports of confirmed symptomatic COVID-19.

Vaccine	Days post-vaccination	Sex			Age Mdn (range)	Previous SARS-CoV-2 infection^∧∧^
		Female	Male	Total		
Pf-BIONT	5	43	19	62	42 (20–63)	16*
	25	37	14	51		
CSBIO	42 (30–57)	16	11	27	45 (23–59)	0

*^∧∧^Recorded symptomatic SARS-CoV-2-like infection.*

*Confirmed by RT-PCR.

*Mdn, median.*

### IgG antibody responses to receptor-binding domain and spike after vaccination

An overall IgG seroconversion of 73% (102/140) was detected against RBD and/or S in all vaccinees. IgG seroprevalence in Pf-BNT vaccinees at 5 dpv against any of the two viral antigens were detected in 55% (34/62) of participants, whereas at 25 dpv, we observed an IgG seroprevalence of 94% (48/51). The percentage of positivity at 5 dpv against the RBD antigen was of 24% (15/62) and 52% (32/62) for S antigen. At 25 dpv the percentage of positivity was of 90% (46/51) against RBD and 90% (46/51) to the S antigen (Table 2).

**TABLE 2:** Number of individuals showing IgG reactivity against the SARS-CoV-2 antigens RBD, S, and N in serum collected from Pf-BNT and CSBIO vaccine recipients.

Vaccine	Days post-vaccination	IgG positivity (*n*) to:				
		Individual viral proteins			Both proteins	
		RBD	S	N	RBD	S
Pf-BIONT	5 (*n* = 62)	15	32	38	13	
	25 (*n* = 51)	46	46	22	11	
CSBIO	42^#^ (*n* = 27)	20	8	12	7	

*^#^Days post-vaccination (dpv) range: 30–57 days.*

In CSBIO vaccinees, the overall seroprevalence was of 78% (20/27), where 74% (20/27) and 29% (8/27) had detectable IgG levels against the RBD and S, respectively (Figures 1A–C). In this group of vaccinees, the time of sample collection varied between 23 up and 57 dpv ($\bar{x}$ = 42 days) (Table 1).

A robust and higher IgG antibody responses against the RBD and S were significantly detected at 25 dpv compared to the early time point of sample collection (5 dpv) in the Pf-BNT group (Figures 1A,B) (Mann–Whitney test, *p* < 0.0001). A multiple comparison analysis showed that regardless of the viral target (e.g., RBD or S), Pf-BNT vaccinees showed higher IgG positive reactivities, particularly after 25 dpv, compared to the CSBIO (42 dpv) (Figures 1A,B). Interestingly, those participants with previous history of COVID-19 before vaccination had high detectable levels of IgG antibodies against the RBD and the S protein of SARS-CoV-2 (Figures 1A,B, *green squares*).

To notice, seropositivity to both antigens, RBD and S, were detected in percentages of 21% (13/62) or 86% (44/51) in Pf-BNT at 5 or 25 dpv, while 26% (8/27) for CSBIO (Table 2).

**FIGURE 2:**
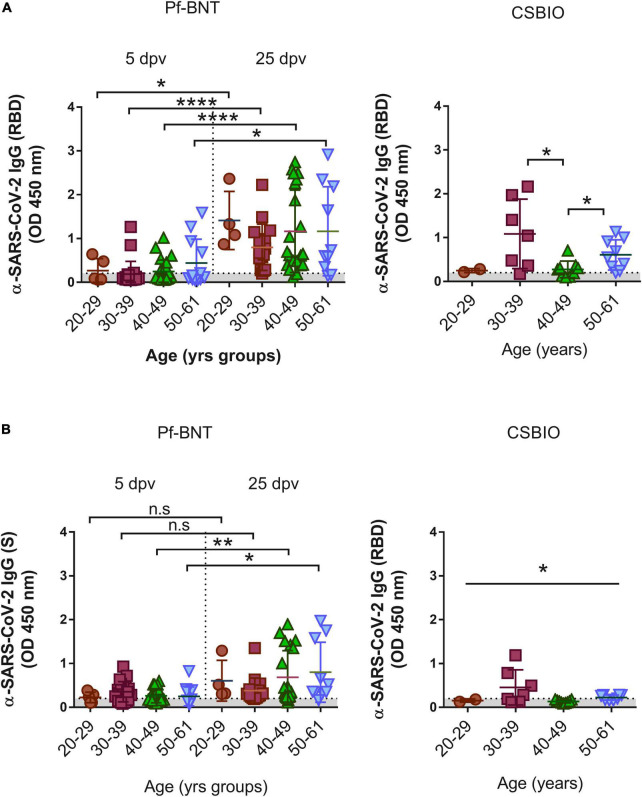
IgG-specific responses to the SARS-CoV-2 RBD by age groups. IgG levels expressed as OD values against the **(A)** RBD and **(B)** S viral targets. A cut-off value >0.20 (horizontal dotted line and *gray zone*, *y* axis) was used to define IgG positive or negative serum samples against SARS-CoV-2 RBD at four age groups, 20–29, 30–39, 40–49, and 50–61 (*x* axis). Serum samples for Pf-BNT (*left panel*) and CSBIO (*right panel*) vaccinees were collected after 5 and 25 (separated by dotted line on *x axis*, *left panel*), and 43 (*n* = 27) dpv, respectively [vaccine values separated by vertical dotted line (*x*-axis)]. Mann–Whitney test: ****p < 0.0001; *p* < 0.001; *p < 0.05. One-way ANOVA (*p* < 0.05). n.s., no significant differences. Each group of data in the scatter plots represent absorbance values (OD) obtained from individual serum sample per vaccine group including the mean ± SEM (standard error) of individual groups.

Finally, we analyzed whether IgG levels against the antigens varies depending on the age at the time of vaccination. In Pf-BNT vaccinees age varied between 20 and 61 years, and for CSBIO vaccinees (20–59 years) (Table 1 and Figure 2). For both group of vaccinees, no significant relationships were found between the age and the IgG levels (OD); however, a *t*-test distribution analyses between age groups, identified significant differences in the IgG levels detected against the RBD between all groups of age (*t*-test *p* < 0.0001) in the Pf-BNT recipients at 5 and 25 dpv (Figure 2A, *left panel*). A similar pattern was identified for the IgG levels detected against the S protein (Figures 2A,B, *left panel*). Regarding the CSBIO vaccine group, significant differences in the IgG levels against the RBD were only detected between age groups of 30–39 and 50–61 years of age when compared to those vaccinees between 40 and 49 years old (*t*-test *p* < 0.05) (Figure 2, *right panel*) while only the group of 30–39 years old had higher levels of IgG against the S protein (Figure 2B, *right panel*).

### Antibody response to severe acute respiratory syndrome coronavirus 2 after natural infection

**FIGURE 3:**
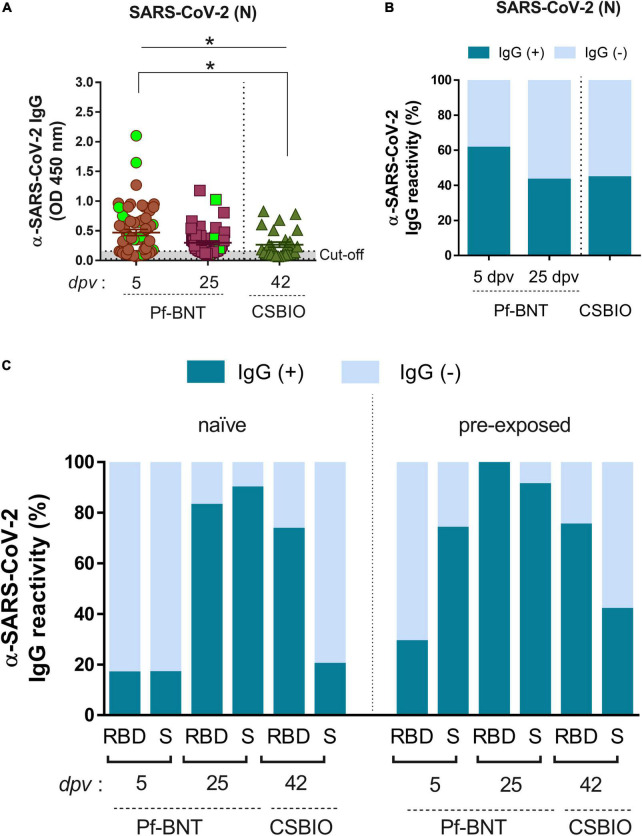
IgG-specific responses to SARS-CoV-2 after natural infection. **(A)** IgG levels expressed as OD values against SARS-CoV-2 N protein determined from positive and negative serum samples based on previously established cut-off values >0.20 (horizontal dotted line and *gray zone*). Serum samples for Pf-BNT and CSBIO vaccinees were collected after 5 (*n* = 62) and 25 (*n* = 51), and 43 (*n* = 27) dpv, respectively [vaccine values separated by vertical dotted line (*x*-axis)]. Mann–Whitney test: *p < 0.05. n.s., non-significant differences. Each group of data in the scatter plots represent absorbance values (OD) obtained from individual serum sample per vaccine group including the mean ± SEM (standard error) of individual groups. Participants with record of COVID-19 (PCR-positive test) before vaccination are highlighted in light green. Stacked bars represent **(B)** total percentage of serum samples per vaccine group showing IgG positive (+, *green*) or negative (–, *light blue*) OD values against SARS-CoV-2 N protein and **(C)** the IgG reactivity to RBD and S in naïve (no-previously exposed) or pre-exposed vaccinees to SARS-CoV-2 infection.

In general, we observed that 53% of all individuals vaccinated with Pf-BNT and 44% vaccinated with CSBIO had reactive IgG antibodies against the N protein (Figure 3A and Table 3). Of these, 42% (16/38) belongs to the previously confirmed COVID-19 group (*green squares*). The remaining 58% (22/38) with anti-N IgG positive ELISA, did not recall being exposed to SARS-CoV-2 before to the vaccine. These results suggest a previous natural infection, possibly asymptomatic. A breakdown of IgG specific reactivity based on the time post-vaccination, shows an observed seroprevalence of 61 or 43% at 5 and 25 dpv in Pf-BNT vaccinees (Figure 3B). Of the sixteen Pf-BNT participants with previous confirmed COVID-19, only 69% (11/16) had IgG seropositivity to N. Of note, 93.75% had IgG reactivity against either RBD or S at 5 dpv (Table 3).

**TABLE 3:** Number of individuals with and without previous exposure to SARS-CoV-2 showing IgG reactivity against the SARS-CoV-2 antigens RBD and S in serum collected from Pf-BNT and CSBIO vaccine recipients.

Vaccine	IgG positivity (*n*)					
	dpv (*n*)	Naïve		dpv (*n*)	Pre-exposed	
		RBD	S		RBD	S
Pfizer/BIONT	5 (*n* = 24)	4	4	5 (*n* = 38)	11	28
	25 (*n* = 29)	24	26	25 (*n* = 22)	22	20
CSBIO	42* (*n* = 15)	11	3	42^#^ (*n* = 12)	9	5

*^#^Days post-vaccination (dpv) range: 30-57 days.*

Based on the N IgG positive results, suggesting previous exposure to SARS-CoV-2, participants were divided into two groups, naïve and pre-exposed. The analysis identified that few Pf-BNT vaccinees (16%) within the naïve group (4/24) seroconverted after 5 dpv against either the RBD or S. At the second sample collection (25 dpv), seroconversion increased significantly to 83% (24/29) against the RBD, and 89% (26/29) to S. Regarding the CSBIO vaccine, 73% seroconverted against the RBD and 20% against the S (Figure 3C and Table 3).

Analyzing the pre-exposed group (anti-N IgG positive), compared to the naïve group, seroprevalence increased for all antigens irrespective of the time of collection or the vaccine composition (Figure 3C and Table 3). Vaccination in the study participants with previous exposure to SARS-CoV-2 infection was positively related to seroconversion against both SARS-CoV-2 antigens, the RBD [odd ratio (OR) = 2.04, 95% CI 0.57, 7.34] and S (OR = 23.83, 95% CI 4.81, 118.17) in the case of Pf-BNT vaccine recipients as well as the RBD (OR = 1.09, 95% CI 0.19, 6.2) and S (OR = 2.86, 95% CI 0.52, 15.77) regarding the CSBIO vaccinees. Our results underline that in both vaccine schemes, IgG seroconversion was positively boosted by previous exposure to SARS-CoV-2 antigens even in asymptomatic infections.

### Severe acute respiratory syndrome coronavirus 2 vaccines induce variable IgG reactivity against receptor-binding domain and spike

**FIGURE 4:**
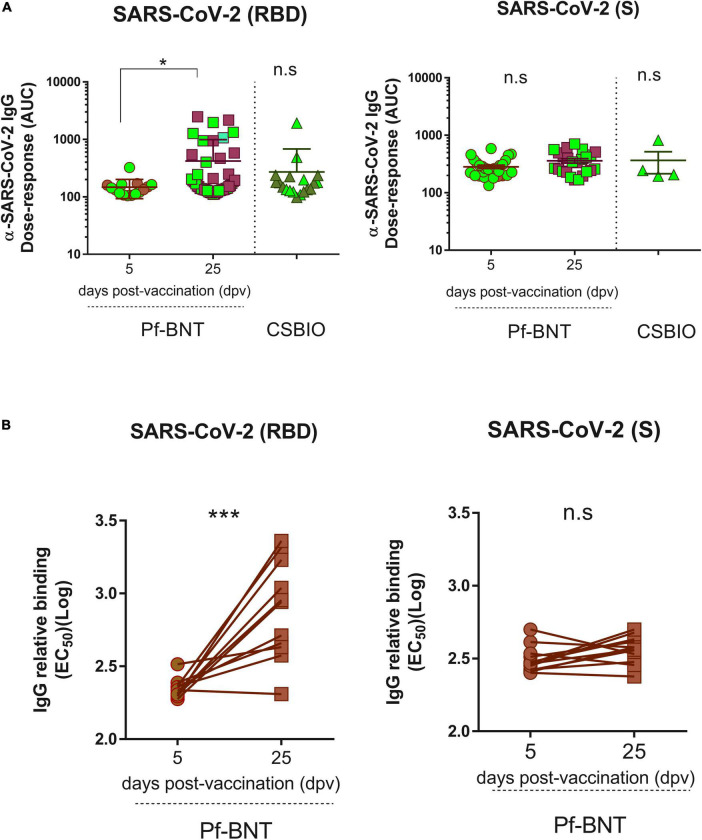
Severe acute respiratory syndrome coronavirus 2 IgG Dose-response against the RBD and the S proteins. An area under curve **(A)** values were estimated from dose response curves with non-linear regression obtained from twofold serially diluted (eight dilutions) IgG positive serum samples against the RBD (*left panel*) and S (*right panel*) proteins of SARS-CoV-2 for both vaccine recipients, Pf-BNT and CSBIO at different dpv. Participants with record of COVID-19 (PCR-positive test) before vaccination are highlighted in light green. **(B)** Comparison between EC_50_ values obtained from paired-serum samples collected after 5 and 25 dpv for Pf-BNT vaccinees against the SARS-CoV-2 RBD (*left panel*) and S (*right panel*) proteins. Mann–Whitney test: *p < 0.05, **p < 0.01; paired *t*-test: ***p < 0.001. n.s., non-significant differences. Each group of data in the scatter plots represent either the area under the curve (AUC) **(A)** or the IgG-binding concentration 50 (EC_50_) **(B)** based on the absorbance (OD) values obtained from individual serum sample per vaccine group against the three SARS-CoV-2 antigens, including the mean ± SEM (standard error) of individual groups.

To follow up our first ELISA screening we further assessed the reactivity (titers) of the IgG antibodies produced in response to the Pf-BNT and CSBIO vaccines. Based on the dose-response curves (Supplementary Figure 2), we could identify those sera collected after 25 dpv from the Pf-BNT group with IgG levels above the cut-off value (*n* = 46) had significantly different IgG reactivity against the SARS-CoV-2 RBD (*p* < 0.0001) compared to sera collected at 5 dpv (*n* = 15) (*t*-test, *p*-value = 0.9991) (Figure 4A). This did not occur when sera were diluted in the presence of S protein, in which no significant variability in the dose-response curves (*p* < 0.9442; *p* < 0.9858) was identified regardless the vaccine preparation (Figure 4A and Supplementary Figure 2). A further comparison between the IgG-specific reactivity (AUC) obtained between the RBD and the S proteins at 25 pdv, showed that the IgG-specific responses to the RBD were significantly different that those obtained against the S protein (*t*-test, *p* = 0.0020) (Figure 4A). These differences were not detected in the CSBIO vaccinees.

Finally, we examined whether the post-vaccination timing affects the IgG levels generated against SARS-CoV-2 RBD and S, after a single-dose of this vaccine (Figure 4B and Supplementary Figure 3). A paired analyses of the EC_50_ values obtained from each seropositive pair of human sera tested against the RBD (*n* = 11), S (*n* = 12), and N (*n* = 13) clearly identified that Pf-BNT vaccinees had a significant increment in the levels of RBD-specific IgG antibodies between day 5 and 25 post-vaccination (*t*-test *p* < 0.0001); not detected against the two other viral targets (*t*-test *p* < 0.0519; *p* < 0.2428). An increment between 0.9- and 11.9-fold in the IgG levels was observed. Noteworthy, more than 80% of Pf-BNT vaccinees with increased levels of IgG had symptomatic and RT-PCR confirmed-COVID-19 infection, which confirm that previous infections with SARS-CoV-2 results in increased IgG responses after vaccination.

## Discussion

In this study, we standardized three serological methods to assess seroconversion against three antigenically important SARS-CoV-2 viral proteins including the RBD, the S, and the N, upon SARS-CoV-2 vaccination in adults of the city of Merida, Mexico. We examined the IgG immune responses of 140 serum samples collected from 89 vaccine recipients. According to their SARS-CoV-2 vaccine schedule, participants had received either a single-dose of Pf-BNT (*n* = 62) or a single-dose of CSBIO (*n* = 27). Our results show an overall seropositivity of 88% after 25 days or more of vaccination, against the RBD and/or the S proteins of SARS-CoV-2. Interestingly, we also detect anti-N IgG antibodies in 51% of all vaccinees, suggesting that those individuals were pre-exposed to SARS-CoV-2 infection before vaccination.

During natural SARS-CoV-2 infection, antibody responses have shown different times for seroconversion against distinct viral antigens depending on the severity of the disease (14–16). The S and N proteins have shown to be highly immunogenic, being the S the main target for neutralizing antibodies (6, 8, 9, 17). Additionally, the RBD within the S represents a critical target when looking at humoral immune responses, as it recognizes the receptor ACE2 specifically mediating virus entry in the cell host (18–20). Here, based on the positive IgG responses detected by our *in-house* ELISA against any of the two SARS-CoV-2 viral proteins, RBD or S, we detected a total seroconversion rate of 90% (46/51) and 74% (20/27) in the Pf-BNT and the CSBIO vaccine groups after 25 and more dpv, respectively. The seroconversion rates in the *naïve* (anti-N IgG negative) populations were 89% in Pf-BNT and 73% in CSBIO vaccine recipients. As expected, seroconversion in SARS-CoV-2 pre-exposed individuals increased up to 100% in Pf-BNT and 75% in CSBIO vaccinees. These findings agree with previous studies showing increased seroconversion efficiencies (around 100%) in Pf-BNT (mRNA-base vaccine) and AstraZeneca (Ad-based vaccine) vaccinees with and without evidence of prior SARS-CoV-2 infection (43). Here, only 16 out of 62 individuals vaccinated with Pf-BNT had a laboratory positive test of COVID-19. Despite this, 56% (26/46) with no record of laboratory diagnosis nor symptomatic infection had anti-N IgG antibodies at 5 dpv. In addition, for the CSBIO vaccinees, none of the participants had a record of previous symptomatic COVID-19 nor laboratory diagnosis. Therefore, the results of pre-exposed vaccinees are a meaningful finding for our study.

In Mexico, vaccination against COVID-19 was implemented following a vaccination scheme based on group of age and risk, first all individuals ≥60 years old with/without comorbidities, and health personnel; followed by all individuals 50–59 years old with/without comorbidities; and then the rest of the Mexican population.^1^ The vaccines available in Mexico are Pfizer-BioNTech, CanSino, COVAX, AstraZeneca, Sputnik V, Sinovac, Janssen, and Moderna. However, in order of number of doses administered, AstraZeneca, CanSino, and Pfizer are the main vaccines in use in Mexico.^2^

Our study analyzed seroconversion in a group of workers with health-related activities who had received only a first dose of Pf-BNT vaccine in April 2021. The second group corresponded to university personnel who were vaccinated with a single dose of CSBIO in May 2021. Unfortunately for this second group, we could only have access to one post-vaccination sample collection (avg. 42 dpv), as samples were collected onside while attending for lab testing at CIR-UADY. Our results consistently showed that the IgG-antibody responses against the RBD expressed as IgG levels (OD values), IgG titers (endpoint dilution), relative binding (EC_50_), and dose-responses (AUC), significantly peaked in the vaccinees after 25 dpv regardless of the vaccine preparation. A similar pattern was detected when S protein was used as target for seroconversion. The IgG levels (OD) against both RBD and S proteins increased in pre-exposed vaccinees, particularly those with confirmed evidence of prior SARS-CoV-2 infection. Our findings are in line with previous reports in which previously SARS-CoV-2 infected vaccinees had higher antibody titers compared with previously uninfected vaccine recipients (44–46). Overall, all these parameters used to assess IgG seroconversion against SARS-CoV-2 were always higher against the RBD compared to the full-length S protein. These results together underline the high immunogenicity induced by the RBD of the SARS-CoV-2 spike protein as previously reported (47–51).

On the other hand, the levels of anti-RBD IgG antibodies detected in both natural SARS-CoV-2 infection and vaccinees have been strongly correlated with the neutralizing capacity of the antibody responses (50, 51). In fact, the RBD has become a major target for therapeutic development as many monoclonal antibodies binding to the RBD can potently neutralize SARS-CoV-2 and have been proposed as potential strategy for effective COVID-19 treatment (22, 51, 52). Regarding the other structural proteins of SARS-CoV-2 such as the N protein, most antibody responses against it has shown not to efficiently neutralize viral infection and so far, few monoclonal antibodies targeting them have been developed. However, they can be used for other applications, such as diagnosis and epidemiology, providing a tool for the early and accurate diagnosis on clinical samples of SARS-CoV-2 (24, 53, 54). One limitation of this study is that we could not evaluate the neutralizing capacity of the anti-SARS-CoV-2 IgG antibodies elicited by the two vaccines, which is critical to understand either the incidence of COVID-19 and the effectiveness of vaccines. This process has been hindered by the lack of a BSL-3 facility used to perform neutralization assays using either wild type virus and/or other *in vitro* approaches (55).

The COVID-19 disease courses from asymptomatic and mild respiratory infections to pneumonia and life-threatening complications such as the ARDS (3, 4). Patients without any symptoms at the screening point are defined as asymptomatic infections, however, can turn into infected people who either develop symptoms later (presymptomatic infections), or never develop any symptoms (true asymptomatic or covert infections) (28–31, 40, 56, 57). In this study, we identified a high percentage of IgG positivity against the viral protein N (56%, 50/89) among the Pf-BNT at 5 dpv and CSBIO at 42 dpv. For both groups of vaccinees, only 16 participants (32%) had history of COVID-19 symptoms, confirmed by PCR laboratory results, which indicates that more than half of these groups of vaccinees went potentially through an asymptomatic SARS-CoV-2 infection.

During the COVID-19 pandemic, several meta-analyses have estimated asymptomatic infections in a wide range, as low as 4% and as high as 80% (28–33). In agreement with these studies, we identified a comparable asymptomatic prevalence of 52% (38/73) in those participants with no history of COVID-19 before vaccination, including Pf-BNT (26/46) and CSBIO (12/27) vaccinees. Here, we could also identify that both anti-N IgG seropositivity as well as the IgG levels (OD values) detected against the N, particularly in the Pf-BNT vaccinees, decayed from >60% (OD mean: 0.4724) to less than 40% (OD mean: 0.2977) after 25 dpv, which indicates that anti-N IgG-specific antibodies were waning at some point.

A limitation of our study is that no basal serum sample was collected before vaccination of the participants and only short-post-vaccination times (less than a month) were examined which hinders the long-term estimations of antibody duration after vaccination. For the Pf-BNT vaccinees, we could collect an early post-vaccination time point at 5 days after the first dose was administrated. This allowed us to identify asymptomatic individuals and differentiate our study population, between *naïve* and pre-exposed, which is a critical aspect to understand the antibody dynamics for SARS-CoV-2. Regarding the CSBIO vaccine, we only had access to post-vaccination samples (42 dpv). Our study and the data reported by Melgoza-Gonzalez et al. (45), are the first results on the antibody IgG response to COVID-19 vaccines in Mexico, including CSBIO, widely used to immunize the education-academic sector (approximately 3.03 million people) even before WHO approval.^3^

In summary, we have demonstrated that vaccination with two distinct vaccine preparations elicited IgG antibody responses that recognized two main targets of SARS-CoV-2, RBD, and S proteins. The ability to accurately detect, measure and characterize the various antibodies specific to SARS-CoV-2 is necessary for vaccine development, manage risk and exposure for healthcare and at-risk workers, and for monitoring reinfections with genetic variants and new strains of the virus. Having a thorough understanding of the benefits and cautions of standardized serological testing at a community level remains critically important in the design and implementation of future vaccination campaigns, epidemiological models of immunity, and public health measures that rely heavily on up-to-date knowledge of transmission dynamics.

## Data availability statement

The raw data supporting the conclusions of this article will be made available by the authors, without undue reservation.

## Ethics statement

The studies involving human participants were reviewed and approved by the Centro de Investigaciones Regionales Dr. Hideyo Noguchi. Universidad Autonoma de Yucatan (CIR-UADY) Protocol number: Record CEI-11-2020. The patients/participants provided their written informed consent to participate in this study.

## Author contributions

GA-T and HP-G: conceptualization and project administration. GA-T, HP-G, MP-C, FP-M, FF-Q, PG-P, SV-J, RG-L, LC-F, JG-C, GV-P, JE, and PM-S: methodology. HP-G, MP-C, FP-M, FF-Q, and GA-T: formal analysis. GA-T, HP-G, MP-C, and FP-M: investigation and data curation. GA-T, HP-G, PM-S, GV-P, PG-P, SV-J, RG-L, and LC-F: resources. HP-G and GA-T: writing – original draft preparation. HP-G, GA-T, MP-C, JE, PM-S, and GV-P: writing – review and editing. GA-T, HP-G, PG-P, SV-J, PM-S, and GV-P: funding acquisition. All authors contributed to the article and approved the submitted version.

## Conflict of interest

The authors declare that the research was conducted in the absence of any commercial or financial relationships that could be construed as a potential conflict of interest.

## Publisher’s note

All claims expressed in this article are solely those of the authors and do not necessarily represent those of their affiliated organizations, or those of the publisher, the editors and the reviewers. Any product that may be evaluated in this article, or claim that may be made by its manufacturer, is not guaranteed or endorsed by the publisher.

## Footnotes

1
http://vacunacovid.gob.mx/wordpress/priorizacion-de-personas-a-vacunar/

2
http://vacunacovid.gob.mx/wordpress/informacion-de-la-vacuna/

3
https://www.who.int/news/item/19-05-2022-who-validates-11th-vaccine-for-covid-19

## Supplementary material

Supplementary Figure 1 Optimization of an *in-house* ELISA for detection of human IgG against SARS-CoV-2 recombinant proteins. **(A)** Non-linear curve fit regression analysis of serially diluted (twofold, eight-dilutions) dose response curves of SARS-CoV-2 viral targets – RBD, S, and the N – to detect human IgG-specific antibodies using an in-house indirect IgG ELISA format. A single dilution of human serum (1:100) along with a combination of anti-human IgG-conjugated to HRP and a colorimetric substrate (TMB) were used to detect the IgG-specific reactivity against SARS-CoV-2 antigens by spectrophotometry (absorbance: OD) at 450 nm. A concentration of 1 μg/mL was determined as the protein assay concentration to coat the ELISA plates. **(B–D)** Determining a cut-off value of >0.20 (horizontal dotted line and *gray zone*) to define IgG positive and negative samples using a reference serum and sera collected in 2016 (*n* = 88) **(B,C)**, and a pool of IgG-positive serum against each SARS-CoV-2 viral proteins **(D)**. Antigen dilution range: 10–0.078 μg/mL used to coat ELISA plates. R square detected for each protein in dose response curves: *R*^2^ = RBD (0.9727); S (0.9573), N (0.9775). Each point represents a geometric mean with 95% confident interval (CI) obtained from three independent experiments. One-way ANOVA *p* < 00.5; non-parametric Student’s *t*-test *p* < 0.05. n.s., non-significant differences.

Supplementary Figure 2 Dose response binding curves of IgG positive sera to the SARS-CoV-2 viral proteins RBD and S. Binding of human sera to the SARS-CoV-2 viral proteins RBD and S was estimated from twofold serially diluted (eight dilutions, range: 1:100–1:12,800) IgG positive serum collected from both vaccine recipients, Pf-BNT and CSBIO at 5 and 25 dpv for Pf-BNT, and 43 dpv for CSBIO vaccinees. Statistically significant differences (*p* < 0.05) were estimated after linear regression analyses within each data points included in the dose response curves obtained by ELISA (OD values) against individual viral antigen and times post-vaccination. Different colors indicate each individual sample. Each group of data in the plot graphs (dots connected lines) represent the relative IgG binding capacity of individual IgG positive serum samples diluted eight times (twofold) starting at 10^–2^. Absorbance values obtained at the dilution of 10^–2^ were considered as the 100% of IgG binding capacity for each individual sample against the SARS-CoV-2 antigen tested.

Supplementary Figure 3 Binding of IgG positive sera to the SARS-CoV-2 viral proteins RBD and S. **(A)** End-point dilution titers and **(B)** EC_50_ values were extracted from dose response binding curves of twofold serially diluted (eight dilutions, range: 1:100–1:12,800) IgG positive serum samples. Mann–Whitney test: **p* < 0.05, ^**^*p* < 0.01, ^***^*p* < 0.001. n.s., non-significant differences. Each group of data in the scatter plots represent Log transformed end-point dilution titers **(A)** and EC_50_ **(B)** obtained from individual serum sample per vaccine group including the mean ± SEM (standard error) of individual groups.
