# Supplementary figures and images for: Human IgG antibody responses to severe acute respiratory syndrome coronavirus 2 viral antigens receptor-binding domain, spike, and nucleocapsid, in vaccinated adults from Merida, Mexico
Source: Front Med (Lausanne). 2022 Jul 22;9:916241. doi: 10.3389/fmed.2022.916241 (PMC9354829; doi:10.3389/fmed.2022.916241)

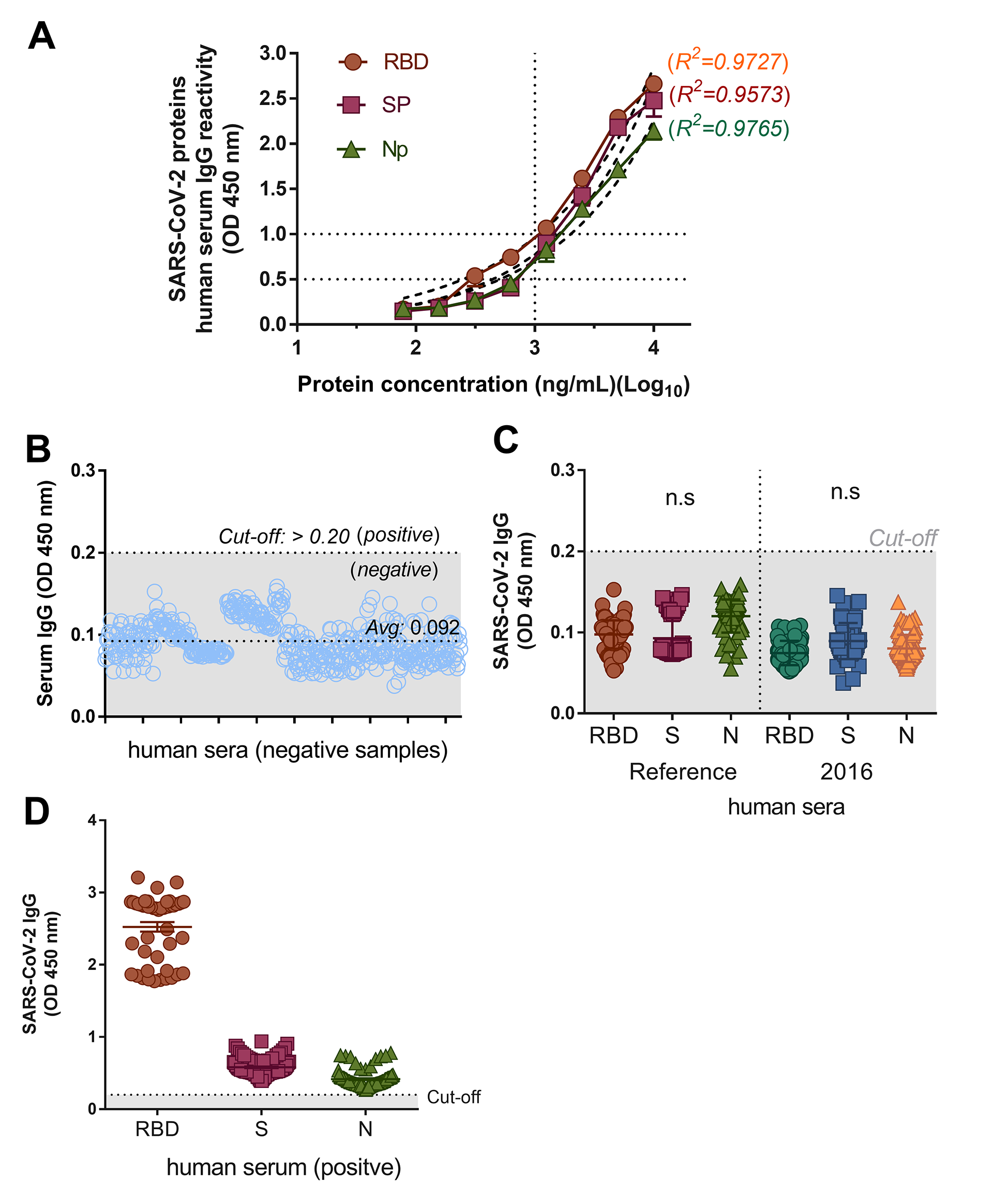

Supplement: Supplementary Figure 1 — Optimization of an in-house ELISA for detection of human IgG against SARS-CoV-2 recombinant proteins. (A) Non-linear curve fit regression analysis of serially diluted (twofold, eight-dilutions) dose response curves of SARS-CoV-2 viral targets – RBD, S, and the N – to detect human IgG-specific antibodies using an in-house indirect IgG ELISA format. A single dilution of human serum (1:100) along with a combination of anti-human IgG-conjugated to HRP and a colorimetric substrate (TMB) were used to detect the IgG-specific reactivity against SARS-CoV-2 antigens by spectrophotometry (absorbance: OD) at 450 nm. A concentration of 1 μg/mL was determined as the protein assay concentration to coat the ELISA plates. (B–D) Determining a cut-off value of >0.20 (horizontal dotted line and gray zone) to define IgG positive and negative samples using a reference serum and sera collected in 2016 (n = 88) (B,C), and a pool of IgG-positive serum against each SARS-CoV-2 viral proteins (D). Antigen dilution range: 10–0.078 μg/mL used to coat ELISA plates. R square detected for each protein in dose response curves: R2 = RBD (0.9727); S (0.9573), N (0.9775). Each point represents a geometric mean with 95% confident interval (CI) obtained from three independent experiments. One-way ANOVA p < 00.5; non-parametric Student’s t-test p < 0.05. n.s., non-significant differences. [file Image_1.tif]

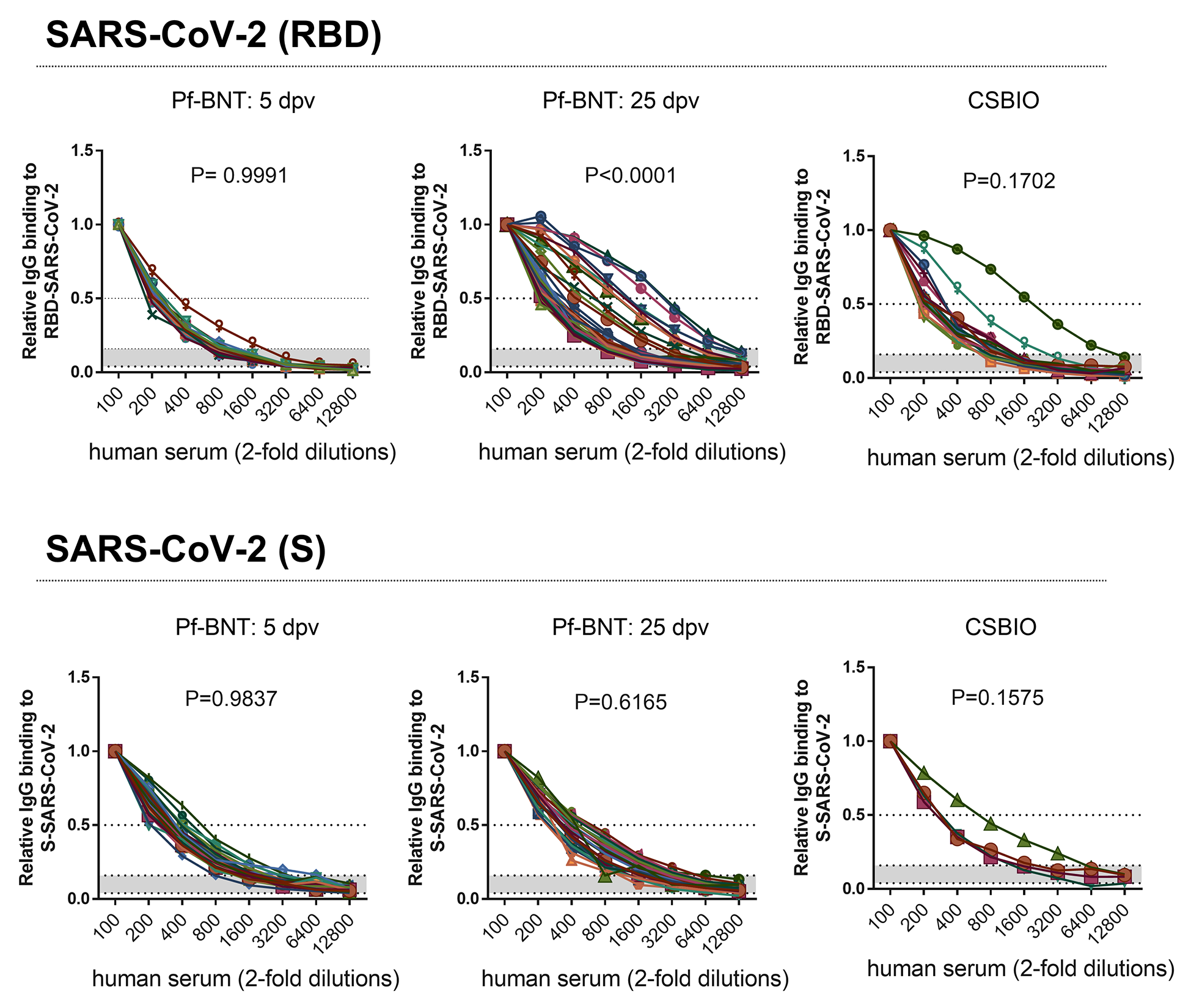

Supplement: Supplementary Figure 2 — Dose response binding curves of IgG positive sera to the SARS-CoV-2 viral proteins RBD and S. Binding of human sera to the SARS-CoV-2 viral proteins RBD and S was estimated from twofold serially diluted (eight dilutions, range: 1:100–1:12,800) IgG positive serum collected from both vaccine recipients, Pf-BNT and CSBIO at 5 and 25 dpv for Pf-BNT, and 43 dpv for CSBIO vaccinees. Statistically significant differences (p < 0.05) were estimated after linear regression analyses within each data points included in the dose response curves obtained by ELISA (OD values) against individual viral antigen and times post-vaccination. Different colors indicate each individual sample. Each group of data in the plot graphs (dots connected lines) represent the relative IgG binding capacity of individual IgG positive serum samples diluted eight times (twofold) starting at 10–2. Absorbance values obtained at the dilution of 10–2 were considered as the 100% of IgG binding capacity for each individual sample against the SARS-CoV-2 antigen tested. [file Image_2.tif]

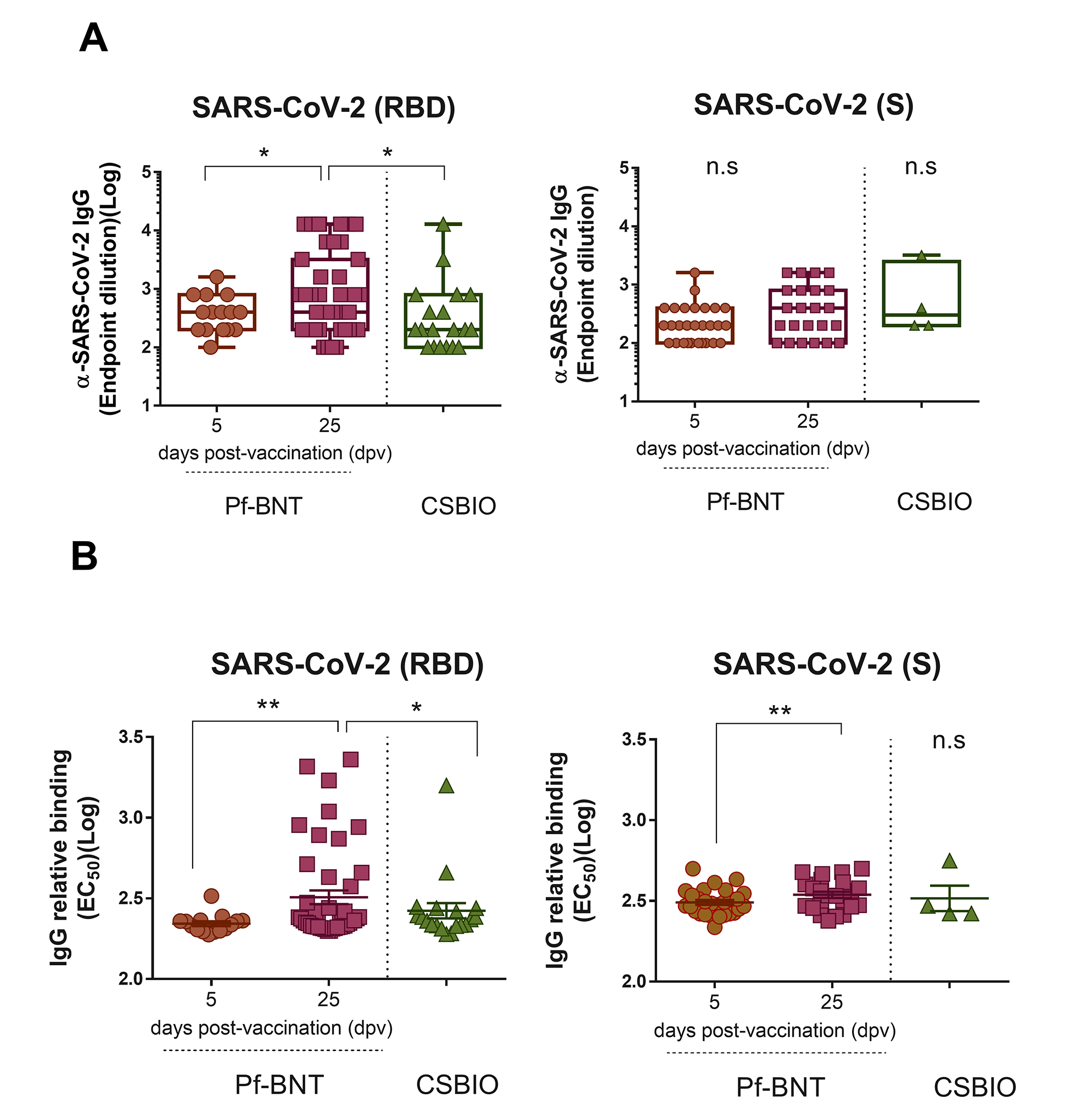

Supplement: Supplementary Figure 3 — Binding of IgG positive sera to the SARS-CoV-2 viral proteins RBD and S. (A) End-point dilution titers and (B) EC50 values were extracted from dose response binding curves of twofold serially diluted (eight dilutions, range: 1:100–1:12,800) IgG positive serum samples. Mann–Whitney test: *p < 0.05, **p < 0.01, ***p < 0.001. n.s., non-significant differences. Each group of data in the scatter plots represent Log transformed end-point dilution titers (A) and EC50 (B) obtained from individual serum sample per vaccine group including the mean ± SEM (standard error) of individual groups. [file Image_3.tif]
